# The Effect of Patient-Specific Instrumentation Incorporating an Extramedullary Tibial Guide on Operative Efficiency for Total Knee Arthroplasty

**DOI:** 10.1155/2017/2034782

**Published:** 2017-08-03

**Authors:** Oh-Ryong Kwon, Kyoung-Tak Kang, Juhyun Son, Dong-Suk Suh, Dong Beom Heo, Yong-Gon Koh

**Affiliations:** ^1^Joint Reconstruction Center, Department of Orthopaedic Surgery, Yonsei Sarang Hospital, 10 Hyoryeong-ro, Seocho-gu, Seoul 06698, Republic of Korea; ^2^Department of Mechanical Engineering, Yonsei University, 50 Yonsei-ro, Seodaemun-gu, Seoul 03722, Republic of Korea

## Abstract

This retrospective study was to determine if patient-specific instrumentation (PSI) for total knee arthroplasty (TKA) leads to shortened surgical time through increased operating room efficiency according to different tibial PSI designs. 166 patients underwent primary TKA and were categorized into three groups as follows: PSI without extramedullary (EM) tibial guide (group 1, *n* = 48), PSI with EM tibial guide (group 2, *n* = 68), and conventional instrumentation (CI) group (group 3, *n* = 50). Four factors were compared between groups, namely, operative room time, thickness of bone resection, tibial slope, and rotation of the component. The mean surgical time was significantly shorter in the PSI with EM tibial guide group (group 2, 63.9 ± 13.6 min) compared to the CI group (group 3, 82.8 ± 24.9 min) (*P* < 0.001). However, there was no significant difference in the PSI without EM tibial guide group (group 1, 75.3 ± 18.8 min). This study suggests that PSI incorporating an EM tibial guide may lead to high operative efficiency in TKA compared to CI. This trial is registered with KCT0002384.

## 1. Introduction

Total knee arthroplasty (TKA) has been established as a reliable treatment for osteoarthritis of the knee [1], and correct alignment of components is fundamental to achieving long-term survival of TKA. A number of studies have suggested that alignment errors > 3° are associated with more rapid failure and less satisfactory functional resources following TKA.

Patient-specific instrumentation (PSI), in which cutting blocks are custom-made for each patient using models based on magnetic resonance imaging (MRI) or computed tomography (CT), was introduced to provide more accurate bone resection and alignment of components and to improve operative room efficiency [2, 3].

Studies have shown that PSI guides used during primary TKA improve operative room efficiency with fewer outliers [3, 4]. In contrast, other data has shown only a marginal improvement in operative room efficiency in PSI compared to similar arthroplasties performed with CI [5, 6]. This difference may be due to a lack of surgical experience and the relatively small number of patients studied to date.

In recently studies, PSI was effective in significantly reducing outliers of optimal rotational femoral and tibial component alignment during TKA [7, 8]. In addition, the stability became improved through the optimization of PSI design. Kwon et al. reported that optimized femoral cutting guide design for PSI showed the closest outcomes in bone resection [9]. In addition, we recently studied that modification of PSI design could lead to shorter surgical time or improved alignment compared with CI [10].

To the best of our knowledge there have been no studies that have evaluated improvements in operative room efficiency and reduced surgical time with respect to tibial guide design utilizing PSI. The hypothesis of this study was that operative room efficiency would be enhanced by using PSI consisting of an extramedullary (EM) tibial guide. Accordingly, the aim of this study was to evaluate the accuracy of bone resections, postoperative tibial component rotations, and posterior tibial slope and particularly to compare operative room efficiency between PSI with and without EM and CI.

## 2. Material and Methods

### 2.1. Patient Enrolment

This retrospective study was approved by the Institutional Review Board of our hospital. Informed consent was obtained from all patients prior to surgery. A total of 166 patients with end-stage knee osteoarthritis scheduled for TKA between November 2013 and February 2015 were included in this study. Patients with rheumatoid arthritis, previous osteotomy, fractures, retained hardware in the limb, or claustrophobia were excluded. The inclusion criteria consisted of a diagnosis of primary knee osteoarthritis and the ability to undergo MRI at our facility. Moreover, patients with defects on the distal femoral or proximal tibial regions who required metal or allograft augmentation or either femoral or tibial stem extensions were excluded because of its influence to the radiographic interpretation for alignment achieved using each surgical technique. All eligible patients were offered to choose between operative options of TKA using PSI and TKA using CI. They were not recommended for any particular surgical technique over the other by a surgeon. In addition, they had been studied from November 2013 to June 2014 for group 1 and from July 2014 to February 2015 for group 2. There were no significant differences in preoperative demographic characteristics and clinical and radiographic data were found between each group, Table 1.

### 2.2. Image Protocol

MRI images were acquired using a 1.5T MRI scanner (Achieva 1.5T; Philips Healthcare, Netherlands). MRI scans were obtained at a slice thickness of 2 mm in the sagittal plane for the tibiofemoral knee joint, while a slice thickness of 5 mm was used for the hip and ankle joints in the axial plane. For nonfat saturation conditions, the MRI consisted of an axial proton-density (PD) sequence. A high resolution setting was used for the spectral presaturation inversion recovery sequence (TE: 25.0 ms, TR: 3,590.8 ms, acquisition-matrix: 512 × 512 pixels, NEX: 2.0, and field-of view: 140 × 140 mm). All procedures were identical to those described for the Signature™ from Biomet (Biomet, Inc., Warsaw, IN, USA).

### 2.3. Presurgical TKA Techniques and PSI Design Methods

**Figure 1:**
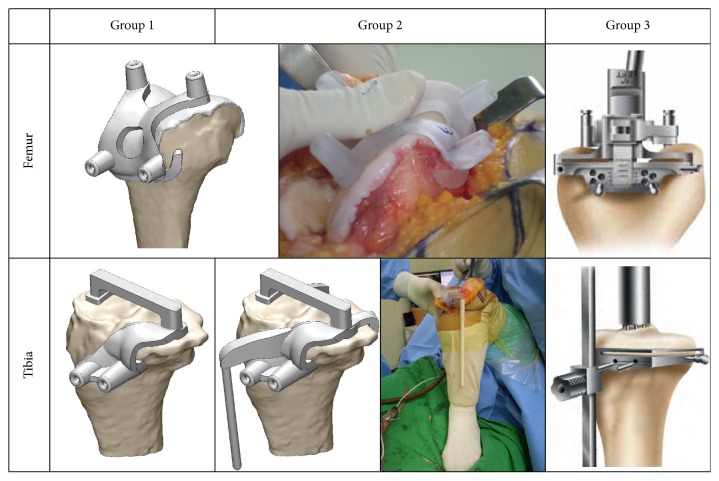
PSI guides and TKA instrument of each group used in this study.

Three-dimensional (3D) data was acquired using MRI scans. The 3D reconstruction process was performed using Mimics software (version 17.0; Materialise, Leuven, Belgium). Using Mimics, the resulting 3D images were converted to STL files and loaded into 3-Matic digital CAD software (Materialise). Importantly, 3-Matic software allows users to combine geometry from mixed sources into a single project. PSI guides were designed in the 3-Matic commercial software (version 9.0; Materialise). We designed and manufactured the initial concept of our PSI on the basis of the Signature™ device, with some differences, because it was found that the rotational stability was important in femoral guide in previous study [9]. For PSI design, femoral guide only restricts the translational stability. Therefore, an additional contact region was developed on the anterior flange in order to produce better rotational stability of the femoral guide (Figure 1). The design of the femoral guides for groups 1 and 2 was identical, with the only contact points in the tibial guide being the proximal tibia and tibial tuberosity. However, for the tibial guide design in group 2, there was an additional contact point placed on the posterior proximal tibia, which included an EM rod (Figure 1). Conversely, a CI system was applied for the control group (group 3) using an EM guidance rod for the tibia and an intramedullary (IM) guidance rod for the femur and spacer blocks [11].

All preplanning and TKA surgeries were performed in our hospital by a single surgeon specialized in orthopaedic surgery (first author). The operating room staff was standardized with two experienced surgeons, surgical scrub technician, physician assistants, and room circulator who had contributed prior to the study. Except for the intraoperative instrumentation, all preoperative management strategies were identical in all three groups. A computer-generated preoperative plan was created according to the following surgeon-specific preferences: default alignment for the femoral component rotation was parallel to the surgical epicondylar axis, the femoral component coronal alignment was 90 degrees to the mechanical axis, and the femoral component sagittal alignment had 3 degrees of flexion with a 9.5 mm distal medial resection length. The default settings for the tibial preparation were perpendicular to the tibial mechanical axis (0° varus/valgus), 8 mm below the lateral plateau high point and 3° posterior slope, and a 90° tibial rotation to the anteroposterior tibial axis (defined as a line connecting the tibial tuberosity tip and the lateral margin of the posterior sulcus) [12, 13]. The slope of the guide device could be changed based on the surgeon's preference. Size and rotation were then adjusted for optimum coverage and posterior fit by estimating the extent of osteophyte removal.

All patients in both cohorts received a posterior-stabilized, fixed-bearing implant. Operations were performed using an anteromedial parapatellar approach without everting the patella. Cement fixation was used in all patients. The Genesis II Total Knee System (Smith & Nephew, Inc., Memphis, TN, USA) was selected as the implant for this study.

### 2.4. Intraoperative and Postoperative Analysis

**Figure 2:**
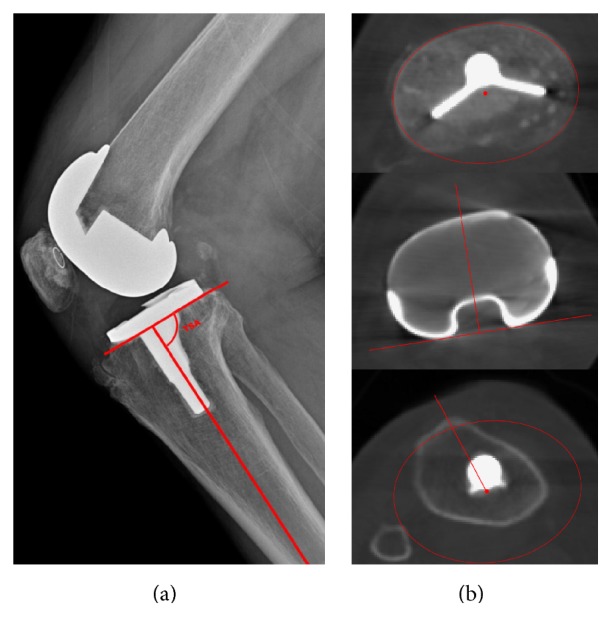
The method for measurement of (a) the tibial sagittal angle and (b) the tibial component rotation angle from CT images.

Surgical time, operative room time, tibial slope, and tibial component rotation outcomes were compared between the PSI and CI groups. The mean value of individual measurements was used for the final calculation of each variable measured in terms of operative efficiency parameters. Resected bone was measured with a 3D laser scanner (Comet VZ; Steinbichler Optotechnik GmbH, Neubeuern, Germany) to an accuracy of 50 *μ*m. The proximal tibia resection was made through the PSI, and the resulting medial and lateral resections were measured for both tibial resections. Bone cutting data was comparatively analyzed with preplanning results. The thickness of the saw blade was added to the resection thickness to calculate the total resection volume. The tibial rotation angle (TRA) was assessed using postoperative CT (Figure 2). The component of tibial posterior slope was measured in terms of the tibial sagittal angle (TSA), on standard lateral radiographs according to techniques described in Figure 2 [14]. Tibial component rotation was measured as described in a recent publication [12, 13], following the technique proposed for CT scans by Berger et al. [15]. Briefly, rotation of the tibial component was determined from three axial slices, namely, just below the tibial base plate, at the level of the polyethylene tibial insert, and at the level of the tip of the tibial tuberosity (Figure 2). The tibial tuberosity axis was plotted from a line connecting the geometrical centre of an ellipse of best fit around the proximal tibia to just below the metal base plate, which was transposed to the image at the level of the tuberosity, and the tip of the most prominent part of the tibial tuberosity itself. Rotation of the tibial component was measured by the angle between the tibial tuberosity axis and a line perpendicular to the posterior edge of the polyethylene insert (Figure 2). Neutral rotation of the tibial component was considered to be 18° of internal rotation from the tibial tuberosity axis [1]. All assessments were performed by two different authors who were not directly involved in the surgical procedures. Each measurement was performed three times at different time points and readers were blinded to the name of the patient and the treating surgeon. Calculations and measurements were performed using digitized images and the previously validated commercial Materialise software [16, 17].

In order to analyze the accuracy of tibial rotation and 3D component positioning between the PSI and CI group, deviations from anatomical rotations and targeted 3D component positioning in degrees were calculated. Outliers were defined as deviations from the intraoperative goals (TSA ± 2°, TRA ± 2°) [18]. Tibial rotation and slope were recorded preoperatively and at the standard 3-month follow-up.

### 2.5. Statistical Analysis

Continuous data were presented as the mean and standard deviation (SD). All statistical analyses were performed in SPSS (version 20.0; IBM SPSS Statistics, Chicago, IL, USA). Two-sample *t*-tests were used to assess differences in operating times between groups. Chi-squared and Fisher's exact test were used to test for significant differences in alignment (proportions) between groups. The level of statistical significance was set at *P* < 0.05.

## 3. Results

Surgical and operative room times were evaluated with respect to the design type of the patient-specific guides compared to group 3 (Table 2). Compared with candidates in group 3, those in the PSI group with EM guide (group 2) had shorter mean surgical and operative room times of 63.9 min (*P* < 0.001) and 97.6 min (*P* < 0.001), respectively. There was no statistically remarkable improvement in surgical time between the PSI without EM guide cohort (group 1) and group 3 (7.5 min saved; *P* = 0.09); however, there was a significant difference in the total operating time (13.2 min saved; *P* = 0.006).

The mean (± standard deviation) differences between the preplanned and actual resection thicknesses are summarized in Table 3 (groups 1-2). The medial and lateral resection errors of the proximal tibia in group 1 were 0.54 mm and 0.51 mm, while those in group 2 were 0.46 mm and 0.49 mm, respectively, the differences of which did not reach statistical significance.

Postoperative radiological evaluation revealed a significant reduction in the outliers and statistical comparisons between study groups for TSA and TRA as shown in Table 4. TSA was found to be decreased in group 2 compared to group 3, and this reduction in group 2 was believed to be due to intraoperative differences in increase of contact points between PSI guide and bone surface, not influenced by significant variations in outliers.

The proportion of patients with TRA malrotation greater than 2° on postoperative analysis of the frontal alignment was 18.0% among CI patients compared with 12.5% in PSI group 1 and 4.4% in PSI group 2 without and with the EM guide, respectively. Overall, the differences between groups 1 and 3 were not statistically significant. On the other hand, the results for groups 2 and 3 with respect to TRA outliers were statistically significant. No patient required revision or presented with a complication from surgery.

## 4. Discussion

To address the expected increase in demand for TKA over the next twenty years, orthopaedic surgeons and medical device manufacturers have developed PSI as a way to provide high quality and efficient patient care at a reasonable cost [19]. There have been relatively few studies that have specifically compared the utility of EM rods and image guidance [20, 21]. Furthermore, to the best of our knowledge, there are currently no prosthesis manufacturers that provide a PSI tibial guide with an EM rod. Thus, the most important finding of this retrospective study of patients undergoing TKA with CI, compared with patients undergoing TKA with PSI with or without an EM guide rod, was that PSI significantly reduced surgical and operative room times.

Ishii et al. [22] found no evidence of a significant difference between alignments achieved using an IM tibial guide and EM guides with standard technique, although they later report that use of an EM guide avoids potential complications of IM guide use, including fat embolization and hypoxia, intraoperative fracture, loss of polymethyl methacrylate pressurization, and inability of IM rod passage due to deformity, retained hardware, or pathologic bone disease [23]. Taken together, the results of these previous studies with those of the present work strongly support our hypothesis that operative room efficiency is enhanced by using PSI with an EM tibial guide.

The available literature is not consistent with respect to the reduction in operative room time with PSI [3, 5, 6, 24–26]. We believe that the reasons for this absence of published information include relatively limited surgical experience and problems in guide design. Indeed, if a surgeon does not have sufficient surgical experience, or even in some cases where they do, mechanical alignment is performed using an EM rod followed by tibial guide pinning. Thus, the main purpose of PSI is to lead to more precise anatomical surgery, better operative efficiency, and fewer surgical complications such as bleeding, infection, and embolism. Therefore, we developed the tibial guide with an EM rod using the validated commercial software Materialise [16, 17]. It is important to note that access to a relatively low cost 3D printer made this work feasible [27].

We believe that the PSI with EM rod described in this study may help inexperienced surgeons gain confidence in PSI applications while also improving operative efficiency. Specifically, the tibial alignment checker rod allowed the surgeon to ensure proper alignment with a proximal tibial cut. Consistently, the EM tibial guide design (group 2) with an alignment checker rod significantly improved surgical time. It could be the reason that there was the preoperative procedure for surgeon to confirm the position of PSI guides with the patient-specific bone model. For group 1, the tibial cutting block was installed in the selected position of pins followed by confirmation of mechanical alignment for tibia with EM tibial alignment guide and ankle clamp. However, in group 2, EM tibial guide was primarily installed and there was no need for additional procedure, which reduced the surgical time and led to improvement in operating room efficiency. In order to obtain more precise measurements, bone resection was measured with a 3D scanner rather than a 2D micrometer, and the resection cutting results measured in this way reflect how well the PSI-TKA surgery was completed after preoperative planning. Resections of the proximal tibia were within 0.54 mm of the predicted value. Thus, we were satisfied with the manner in which the PSI allowed surgeons to cut as planned (groups 1 and 2).

A risk of early loosening due to inconsistent stress distribution may be induced by malrotation of the tibial component and unbalanced patellofemoral joint kinematics, which likely leads to patellofemoral instability and pain accompanied by internal rotation of the tibial component [28]. However, there have been only a few studies conducted with respect to rotation of the tibial component [12, 13]. For example, Silva et al. reported the effect of PSI-TKA on malrotation, and failed to observe a statistically significant difference compared to TKA performed with CI. On the other hand, PSI-TKA led to decreased dispersion and amplitude of the tibial rotation component [13]. In addition, Silva et al. found that use of MRI is associated with decreased malrotation compared to CT imaging. Together, these results were in good agreement with those of the present study. Specifically, normal PSI design in group 1 produced less TRA outliers compared with group 3 in which CI was used, although the difference was not statistically significant. In contrast, the results for group 2 in which the PSI had an additional contact point on the posterior proximal for improving stability resulted in better outcomes in terms of the number of outliers compared to group 3 in which CI was used. It showed the similar trend in posterior tibial slope with tibial rotation. This difference could be caused by instability in drilling. For group 1, there were contact points only in anterior and superior of tibial PSI guide. Furthermore, the stiffness of plastic material used in PSI guide was lower than that of metallic material used in tibial cutting block. Therefore, in drilling, the stability of PSI guide could be reduced. It was due to the additional contact point placed on the posterior proximal tibia and it led to more stable tibial PSI guide fixation.

This observation supported the possibility of using the novel PSI described in this study to enhance surgical outcomes and operative efficiency in PSI-TKA. In other words, the effect of presence of EM guide and additional contact point on tibial PSI guide were to improve operative efficiency and stable fixation, respectively. These results also highlighted the positive future of 3D printing technology in medical applications.

There were several limitations to the present study as follows. First, the study did not include long-term clinical outcomes. Second, the current study consisted of a nonrandomized design, as group allocation was based on patient preference. Thus, further rigorous comparative research may be necessary to obtain more substantive results. Third, we only compared operating times and medical image measurements in determining cost/benefit ratios and did not take into consideration clinical results or patient satisfaction scores. Finally, the number of patients for PSI in groups 1 and 2 has been progressively collected; thus the learning curve could have influenced our results.

Although it is important to highlight the potential limitations of this study, it appears that our results were slightly more favorable than the currently available literature with respect to PSI techniques and their performance.

In conclusion, our analysis revealed that small changes in PSI design, even those considered to be minor, may afford considerable reduction in surgical time and improvement in tibial component alignment compared with CI for TKA. If future studies can validate these advantages, then PSI may reflect the modern standard of surgical care in knee arthroplasty. Further studies should be conducted with larger cohorts and longer follow-up periods to confirm our findings.

## Figures and Tables

**Table 1:** Patient demographics.

	Age	Gender (female/male)	BMI	Mechanical tibiofemoral angle
Group 1 (*n* = 48)	73 ± 4.3	46/2	28.1 ± 4.7	Varus 8.9 ± 5.7°
Group 2 (*n* = 68)	74 ± 7.1	62/6	27.9 ± 4.8	Varus 8.8 ± 5.1°
Group 3 (*n* = 50)	72 ± 6.5	47/3	28 ± 5.1	Varus 8.6 ± 6.3°
*P* value (group 1 : group 3)	0.38	N/A	0.91	0.80
*P* value (group 2 : group 3)	0.11	N/A	0.75	0.85

(N/A, not available; BMI, body mass index).

**Table 2:** Comparison of surgical time, operative room time, and blood loss from each group.

	Surgical time (min)	Operative room time (min)	Estimated blood loss (mL)
Group 1 (*n* = 48)	75.3 ± 18.8	115.5 ± 23.7	109.2 ± 58.4
Group 2 (*n* = 68)	63.9 ± 13.6	97.6 ± 14.9	105.4 ± 54.9
Group 3 (*n* = 50)	82.8 ± 24.9	128.7 ± 22.9	125.9 ± 97.5
*P* value (group 1 : group 3)	0.09	0.006	0.304
*P* value (group 2 : group 3)	<0.001	<0.001	0.18

**Table 3:** Thickness differences of proximal tibia between the preplanned and actual resection.

	Medial	Lateral
Group 1 (*n* = 48)	0.54 ± 0.24 mm	0.51 ± 0.21 mm
Group 2 (*n* = 68)	0.46 ± 0.29 mm	0.49 ± 0.19 mm
*P* value (group 1 : group 2)	0.107	0.6

**Table 4:** Comparison of outliers on tibial posterior slope and tibial rotation angles between PSI and CI after TKA.

	Group 1 (*n* = 48)	*P* value (group 1 : group 3)	Group 2 (*n* = 68)	*P* value (group 2 : group 3)	Group 3 (*n* = 50)
TSA	5	0.56	2	0.04	8
TRA	6	0.71	3	0.03	9
